# Effects of corn distillers dried grains on dough properties and quality of Chinese steamed bread

**DOI:** 10.1002/fsn3.1604

**Published:** 2020-06-16

**Authors:** Xiaona Li, Chunyang Wang, Padmanaban G. Krishnan

**Affiliations:** ^1^ College of Food Science Shenyang Agricultural University Shenyang China; ^2^ College of Health and Human Sciences North Carolina State University Cullowhee NC USA; ^3^ Department of Dairy and Food Science College of Agriculture, Food and Environmental Sciences South Dakota State University Brookings SD USA

**Keywords:** C‐Cell, Chinese steamed bread, corn distillers dried grains, Mixolab, quality analysis

## Abstract

Chinese steamed bread (CSB) accounts for 30% of the wheat end‐use in China. CSB was studied as a platform for fiber and protein enrichment, employing corn distillers dried grains. Food grade distiller's grain (FDDG) processed from co‐products from the corn ethanol industry was used as the enrichment ingredient. Since CSB uses a lean formula with little or no added sugar or fat, it relies entirely on fermentation and steaming for flavor and texture development. FDDG was used to replace 0%–25% all‐purpose flour (APF) in CSB formulations. Effects of FDDG on dough properties and quality of CSB were evaluated by instrumental (Farinograph, Mixolab, and Texture Analyzer), nutritional, and sensory methods. Protein and dietary fiber contents showed significant increases to 18.8% and 15.3%, respectively, for 100 g of steamed bread (25% FDDG db). Fiber in 100 g of fresh FDDG CSB ranged from 2.8 to 7.7 g. FDDG fortified doughs demonstrated higher water absorption, while dough development time, dough stability, and dough extensibility decreased significantly with partial APF replacement. FDDG contributed to increased hardness and adhesiveness in the CSB. Crumb analysis revealed reduced number of gas cells at higher FDDG substitution. FDDG enrichment reduced brightness (L*) of flour blends and CSB. Rheological and sensory analysis showed an upper level of FDDG substitution of 15% was acceptable without detriment to dough functionality, texture, and taste.

## Graphical abstract

This study provides compositional and food functional attributes of Chinese steam bread fortified with a high protein and high fiber ingredient. Distillers dried grains (38% protein and 40% dietary fiber) from corn was developed for food‐grade application and enrichment of steamed bread.

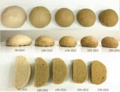

## 1 INTRODUCTION

Steamed bread is a traditional staple food in China with 1,700 years of history. The main ingredients of Chinese steamed bread (CSB) are wheat flour, yeast, and water. CSB accounts for 30% of wheat staple food in China (Jin et al., 2013; Liu, 2014). A survey in Shanghai showed that the average CSB consumption was one per 15 people in the 30 million population. In Hebei Province, consumption rate was one steamed bread per capita per day (Liu, 2014). CSB made with corn, black rice, and wheat flour is popular in China. Typically, steamed bread has an elastic, chewy, spongy, and uniform internal texture, along with a smooth and shiny surface. Processing techniques for CSB are different from conventional oven baked bread. Lower heat used in steam processing (100°C) may reduce nutrient loss in comparison to conventional oven baking. Conventional bread baked at 200°C leads to losses of lysine and vitamin B1. The protein efficiency ratio (PER) of steamed bread is higher than that of baked bread. Oven‐baked bread is made using flour, salt, sugar, and fat. In contrast, the energy contained in CSB, with its leaner formula, is significantly lower than that of baked bread made with the same amount of flour. Steamed bread is thus more suited for diabetic diets. CSB, made with refined wheat flour, needs further improvement. Fortification with adjuncts such as distillers dried grain (DDG) shows potential for nutritional enhancement of conventional foods. Other workers have used okara, corn, rice bran, and wheat bran to replace part of the wheat flour in bread or steamed bread to improve the nutritional value of products (Liu, Singh, & Inglett, 2011; Lu, Cui, Liu, & Li, 2013; Rose, Inglett, & Liu, 2010).

Corn DDGs are co‐products of the corn ethanol industry. The annual output of DDG is large (39 million MT in 2014). DDG serves predominantly as animal feed and is thus an underutilized, yet valuable resource (Stein & Shurson, 2009). Development of new applications for DDG has significant nutritional and economic benefits. DDG is an ideal source of insoluble dietary fiber (35% NDF), comprising of cellulose, hemicellulose, and lignin (Rose et al., 2010). Dietary fiber has a beneficial effect in the prevention of coronary heart disease, colon cancer, obesity, and diabetes (Kaczmarczyk, Miller, & Freund, 2012). Adding DDG to wheat flour may not only add value to a corn co‐product, but it can also improve the nutritional profile of wheat flour (Rosentrater & Krishnan, 2006).

Wheat flour, which is the principle ingredient of CSB, influences the quality of CSB (Wang, Hou, & Dubat, 2017). Optimal DDG substitution of wheat flour in bread formulations can change the quality of traditional baked bread owing to high levels of protein, dietary fiber, and pigments (Pourafshar, Rosentrater, & Krishnan, 2014a). In the present study, rheological behavior of the flour, image analysis, texture profile analysis, and quality analysis of steamed bread were determined to evaluate the effects of DDG on the quality of steamed bread. Two groups have reported on the rheological, texture profile analysis, and sensory properties of dough and steamed bread substituted with various dietary fibers. Fu, Chang, and Shiau (2015) reported that the addition of 3%–6% lemon fiber increased the hardness of steamed bread and decreased cohesiveness, specific volume, and elasticity. Wu and Shiau (2014) investigated the effects of different amounts of pineapple peel fiber on rheological and textural properties of dough and steamed bread. They determined that steamed bread with 5%–10% pineapple peel fiber increased dietary fiber. Liu et al. (2011) reported that traditional corn bread with 30 g/100 g DDGS reduced textural quality. Tsen, Weber, and Eyestone (1983) and Pourafshar et al. (2014a), Pourafshar, Rosentrater, and Krishnan (2014b) used DDGS in conventional bread and tortilla production, respectively. There is a paucity of information on CSB made using corn DDG as a high fiber and high protein ingredient. Results from our study provide a scientific foundation for expanded use of DDG and enhancement of fiber and protein content of CSB.

## 2 MATERIALS AND METHODS

### 2.1 Materials

Distillers dried grain samples obtained from a commercial fuel ethanol plant were stored frozen at minus 80°C until further refinement. All‐purpose flour (APF) and active dry yeast, obtained from commercial sources locally, were stored frozen.

### 2.2 Methods

#### 2.2.1 DDG preparation for food applications

Food grade DDG specific for this study was prepared using solvent extraction, multiple washing, drying, and fine‐grinding protocols developed in our university. A Retsch Mill (GmbH and Co.KG, 5657 HAAN1) operated at 20,000 rpm and with a 0.5‐mm sieve, ensured fine milling. DDG was also steam‐sterilized and controlled for particle size. Two‐kilogram batches of DDG/APF blends were prepared for each of the four treatments (10%, 15%, 20%, and 25% DDG fortification levels). A control flour sample made up of 100% all‐purpose wheat flour served as 0% DDG level. Samples were stored at 4°C. Several researchers have reported on the chemical composition of DDG and its food functionality traits (Pourafshar et al., 2014a, 2014b;Rosentrater & Krishnan, 2006; ; Singha, Muthukumarappan, & Krishnan, 2018; Singha, Singh, Muthukumarappan, & Krishnan, 2018).

#### 2.2.2 Flour blends preparation

A twin‐shelled blender facilitated preparation of DDG and APF blends (Peterson Kelly Co. Inc). This instrument, operated for 1 hr, accomplished homogenous distribution of DDG within the wheat flour. Thus, four types of blends (10%, 15%, 20%, and 25% food grade distiller's grain [FDDG] in APF) were prepared along with the 100% APF.

#### 2.2.3 Physicochemical analysis

AACCI Method 44‐15 was used for moisture content determination. Incineration at 550°C (AACC Method 08‐01) yielded ash content of samples. Protein analysis employed a combustion method according to AACC Method 46‐30 (N × 6.25). Fat content analysis was accomplished by refluxing samples with petroleum ether (AACC Method 30‐25.01). Neutral detergent fiber (NDF) was determined according to AOAC Method 30‐25. Total dietary fiber (TDF) content was determined using the Megazyme enzymatic procedure (AACC Method 32‐07.01). Color parameters were recorded using a Minolta Colorimeter (Minolta Camera Co, Ltd.) using the Hunter L*, a*, and b* color scale.

#### 2.2.4 Preparation of steamed bread

There were five treatments reflecting five levels of fiber substitutions in wheat flour (0% DDG served as the control). Five flour blends were produced (0%, 10%, 15%, 20%, and 25% DDG/APF). Each treatment received three separate bread‐making tests. This means that each blend yielded three 200 g dough pieces. Each 200 g dough piece, which was further subdivided into yield two portions of 100 g bread dough, went through the final steaming process. Therefore, each treatment was represented by six loaves of steamed bread. In total, 30 loaves of steamed bread were prepared to study the effects of five levels of DDG substitution in all‐purpose flour. This level of replication is adequate for determination of effects of fiber and protein fortification on nutritional composition and sensory quality of steamed bread.

Steamed bread with and without DDG was prepared as prescribed by Chinese Standard GB/T17320‐2013. Rheological analysis using the Mixolab provided information on the amount of water needed for production of optimum dough from each flour. Dry yeast (2 g, 1%), dissolved in water (30°C), was stored in a proofing cabinet. For each of the flour types (Control APF and 4 DDG/APF blends), 200 g of flour was added to the bowl along with the yeast solution and salt and mixed in a Kitchen Aide type mixer for 6 min until the gluten formed and the dough surface smoothed out. Each dough piece was passed repeatedly (15–20 times) between the rolls of a dough sheeter set at 0.6‐mm roller gap, and the sheets were then rolled into cylinders and each was divided into two pieces. The separated dough was shaped by hand to yield a smooth dough ball, placed in a steamer tray lined with wet cheesecloth and then proofed for 40 min at 37°C and 75%–80% RH. A stainless steel steamer facilitated the steaming of the dough pieces for 15 min. The baking trials thus involved six replications of steamed bread, triplicate flour blends from each treatment, and 30 loaves of steamed bread to insure proper statistical representation and discerning of effects of experimental variables on steamed bread quality and nutritional composition.

#### 2.2.5 Mixolab analysis

Rheological behavior of dough was determined with the Mixolab^®^ (Chopin Technologies). The “Chopin+” protocol included an initial equilibration at 30°C for 8 min, heating to 90°C at a rate of 4°C/min and holding at 90°C for 7 min, cooling to 50°C at a rate of 4°C, and then holding at 50°C for 5 min. Mixing speed was 80 rpm. Parameters obtained from a typical Mixolab curve were peak mixing time, absorbance, and dough stability (Chakraborty, Tiwari, Mishra, & Singh, 2014; Hadnadev, Torbica, & Hadnadev, 2011; Rosell, Marco, García‐alvárez, & Salazar, 2011).

#### 2.2.6 Dough extensibility

Dough extensibility and resistance to extension were determined using the Texture Analyzer (TA‐XT2i, Stable Micro Systems) equipped with a Kieffer dough and gluten extensibility rig (A/KIE) and a 5‐kg load cell operated in tension mode. Ten‐gram dough balls placed onto the oiled grooved mold were pressed into dough strips. Following a 45‐min gluten resting stage, dough strips were stretched until they fractured. Kieffer measurements employed the following settings: pretest speed: 2.0 mm/s, test speed: 3.3 mm/s, post‐test speed: 10.0 mm/s, distance: 75 mm, trigger force: auto–5 g, and data acquisition rate: 200 pps (Fu, Shiau, & Chang, 2014; Heitmann, Zannini, & Arendt, 2015; Wu, Wang, Li, & Qu, 2014).

#### 2.2.7 Texture profile analysis

Texture profile analysis (TPA) of steamed bread was performed using a Texture Analyzer (TX.XT‐Plus, Stable Micro Systems) equipped with a 5‐kg load cell and a 25‐mm‐diameter cylindrical probe (P/25). The TPA of steamed bread was accomplished 24 hr after steaming. Steamed breads sliced at their largest circumference yielded uniform slices of 25 mm thickness. The texture analyzer employed a pretest speed of 1 mm/s, test speed of 1 mm/s, post‐test speed of 2 mm/s, a compression strain of 40%, and an auto trigger force of 5 g (Lu et al., 2013).

#### 2.2.8 Image analysis

Image analysis of steamed bread was determined 24 hr after steaming using a C‐Cell Bread Imaging System and C‐Cell version 2 Software (Calibre Control International Ltd.). The C‐Cell yielded information on slice area, slice brightness, number of cells, cell diameter, and wall thickness (O'Shea, Rößle, Arendt, & Gallagher, 2015).

#### 2.2.9 Quality analysis of steamed bread

**FIGURE 1:**
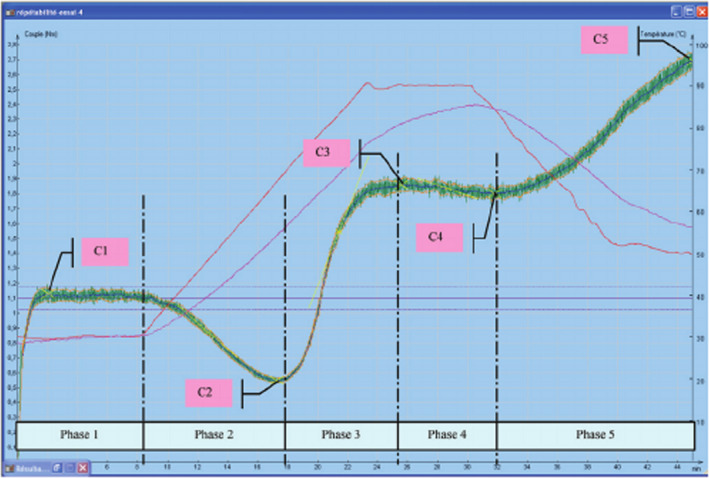
A Mixolab curve showing mixing profile of wheat flour

A sensory panel scored the steamed bread after reheating in the steamer (6–8 min) and cooling for 3–5 min. Thirty loaves of steamed bread were prepared representing five treatment flour blends. Six steamed bread loaves represented each of the five treatments. The quality scoring system was set up according to China Standard GB/T 17320‐2013 (Table 1). Height of streamed bread was determined using a Vernier caliper. The volume of steamed bread was recorded using rapeseed displacement (AACCI Method 10‐05.01). The steamed bread samples placed in sampling cups were coded using random numbers and presented to a trained panel of judges (six males and six females). This panel evaluated samples of steamed bread containing 10% DDG, 15% DDG, 20% DDG, and 25% DDG, including a control (0% DDG). Investigators replicated sensory analysis to insure that there was consistency in their responses. Panelists evaluated the steamed breads for acceptability of the specific volume, height, surface color, surface structure, exterior appearance, interior structure, elasticity, chewiness, stickiness, and flavor (Figure 1). An overall total score based on 100 points was used to rate the steam bread as excellent, good, average or acceptable, and poor.

**TABLE 1:** Quality scoring system for steamed bread fortified with distillers dried grains

Quality parameter	Full score	Scoring criteria
Specific volume (volume/weight, ml/g)	15	Specific volume ≥ 2.8 = 15; Specific volume ≤ 1.5 = 2; 2.8 ≤ Specific volume ≤ 1.5, minus 1 score per 0.1 ml/g reduction
Height	5	Height ≥ 7 cm = 5; Height ≤ 5 cm = 1; 5 cm ≤ Height ≤ 7 cm, minus 1 score per 0.5 cm reduction
Surface color	10	White, milky white = 8–10; Light yellow, yellow = 6–8; Gloom = 2–6
Surface structure	10	Smooth = 8–10; Uneven, pliable, air bubble or concave = 3–8
Exterior appearance	10	Symmetrical, spherical = 7–10; Flat, unsymmetrical = 4–7
Interior structure	15	Tiny pores, uniform = 12–15; Close pores, uniform or separated between the edges and the epidermis = 8–12; Large pores, rough structure = 5–11
Elasticity	10	Rebound quickly, can be compressed more than 1/2 = 7–10; Rebound slowly or does not rebound = 3–7; Difficult to compress, very hard = 2–6
Chewiness	10	Chewy = 7–10; Tender, crumble, low elasticity = 4–7
Stickiness	15	Does not stick to teeth = 8–10; Slightly sticky or sticky = 3–7
Flavor	5	Wheat flavor, no peculiar flavor = 4–5; Bland flavor = 3–4; Peculiar flavor = 1–3
Total score	100	—

Note

Scoring system: poor = <70%, average or general or acceptable = 70–79, good = 80–89, excellent = 90–100.

#### 2.2.10 Scanning electron microscopy (SEM)

A scanning electron microscope (SEM; S‐3400N, Hitachi Co.) enabled the study the crumb structure of steamed bread. Freeze‐dried steamed bread (0.5 cm^3^) was mounted on circular aluminum stubs with double‐sided tape and coated with gold prior to electron microscopy (Kim, Morita, Lee, & Moon, 2003).

#### 2.2.11 Statistical analysis

All data were determined in triplicate and expressed as mean ± standard deviation. A one‐way analysis of variance (ANOVA) and Duncan's multiple range tests enabled determination of significant difference of means at *p* < .05 employing SPSS software.

## 3 RESULTS AND DISCUSSION

### 3.1 Physicochemical properties

**FIGURE 2:**
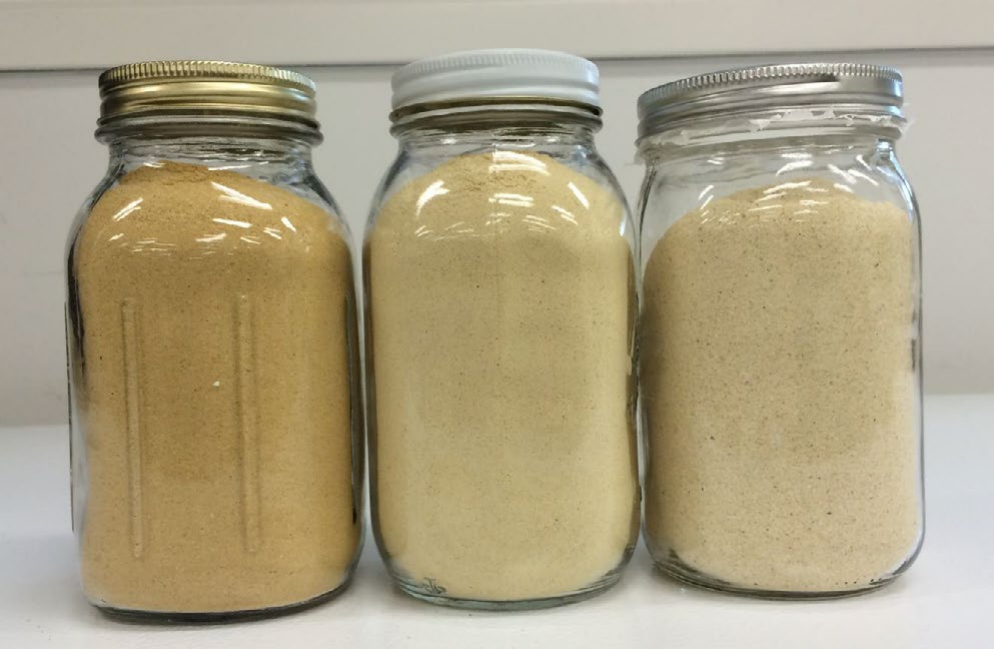
Unprocessed DDG (left), processed ground DDG (middle), and unground processed DDG (right) used for enrichment of wheat flour (DDG, distillers dried grain)

Table 2 provides the composition of starting materials used in the production of CSB. Food grade DDG was predominantly protein (31.9%) and fiber (43.3%) in composition. Figure 2 provides images of raw and processed DDG. Color measurements, as represented by L*, a*, and b* values, showed DDG to be darker (*L* = 86.45) than APF (*L* = 91.43).

**TABLE 2:** Physicochemical properties of food‐grade DDG and all‐purpose flour

Flour type	Moisture (%)	Protein (% db)	TDF (% db)	Fat (% db)	Ash (% db)	Color		
						L*	a*	b*
All‐purpose flour	11.8	10.4	2.85	1.05	0.41	91.43	0.13	8.14
FDDG	6.9	31.9	43.3	2.16	3.16	86.45	−0.31	19.72

Abbreviations: FDDG, food‐grade distillers dried grain; TDF, total dietary fiber.

Proximate analysis of control and DDG‐containing steamed bread (Table 3) showed that the moisture, protein, and fiber content of steamed bread increased (over control) with increasing amounts of DDG in the formula. The protein content of 25% DDG steam bread was 18.8%, significantly higher than that of the unfortified control (12.3% protein). Flour replacement with 10, 15%, 20%, and 25% DDG yielded total dietary fiber (%TDF) content of 2.77, 4.81, 5.87, and 7.71 g of fiber, respectively, for 100 g of steam bread consumed. Protein content also showed a linear increase from 12.3% in the control to 18.8% in the 25% DDG steamed bread. Fat in steamed bread remained below 0.7%. CSB is essentially a low‐fat food product based on the formulation. As 15% and 20% DDG steamed breads did not show significant differences in protein or fiber content, the upper fortification limit could be placed at 15% DDG. This is a modest fortification level while retaining sensory and food functional traits similar to the control. Table 4 provides data on the color of DDG–flour blends and steamed bread made with them. The addition of DDG significantly and consistently decreased the brightness (L*) of DDG–flour blends. With the increasing amount of DDG, the yellowness (b*) of the blends and steamed bread increased due to the pigments originating in the corn. Increasing levels of DDG in the formulation resulted in significant redness in the steamed breads. Liu et al. (2011) reported that DDGS in cornbread induced a decrease in brightness (L*) and yellowness (b*), as well as an increase in redness (a*). These results were consistent with the findings of Tsen et al. (1983), Rasco, Downey, and Dong (1987), Singh, Liu, and Vaughn (2012), and Pourafshar et al. (2014a).

**TABLE 3:** Proximate analysis of Chinese steamed bread enriched with distillers dried grains

DDG (%)	Moisture (%)	Protein (% db)	NDF (% db)	Fat (% db)	TDF^†^ (% db)	TDF^†^ (% wb)
Control	44.5 ± 3.68 a	12.3 ± 0.89 a	0.57 ± 0.02 a	0.37 ± 0.02 a	2.85 ± 1.09 a	1.58 ± 0.60 a
10%	47.0 ± 2.19 b	15.2 ± 0.62 b	4.91 ± 0.10 b	0.40 ± 0.01 a	5.22 ± 0.14 a	2.77 ± 0.07 a
15%	48.1 ± 0.21 cb	16.3 ± 0.71 cb	6.67 ± 0.03 c	0.26 ± 0.03 b	9.26 ± 0.66 b	4.81 ± 0.35 b
20%	48.7 ± 0.64 bd	17.5 ± 0.71 ac	9.78 ± 1.27 d	0.65 ± 0.00 c	11.45 ± 1.07 b	5.87 ± 0.54 b
25%	49.5 ± 0.49 d	18.8 ± 0.49 d	11.72 ± 0.46 e	0.29 ± 0.01 b	15.28 ± 2.38 c	7.71 ± 1.20 c

Note

Means ± standard deviation values in the same column that followed by different letters are significantly different (*p* < .05).

Abbreviation: NDF, neutral detergent fiber.

^†^ TDF: total dietary fiber as consumed in a 100 g portion of steamed bread.

**TABLE 4:** Color of DDG/APF flour blends and steamed breads

Sample	DDG/APF flour blend color			Steamed bread color		
	L*	a*	b*	L*	a*	b*
Control	91.43 ± 0.20 a	0.13 ± 0.01 a	8.14 ± 0.13 e	76.83 ± 0.17 a	−0.62 ± 0.07 e	13.54 ± 0.12 c
10% DDG	90.06 ± 0.11 b	−0.16 ± 0.04 b	11.91 ± 0.11 d	72.50 ± 0.15 b	−0.05 ± 0.02 d	19.15 ± 0.16 c
15% DDG	89.87 ± 0.06 b	−0.26 ± 0.02 c	12.92 ± 0.20 c	69.62 ± 0.15 c	0.36 ± 0.01 c	19.83 ± 0.35 b
20% DDG	89.39 ± 0.08 c	−0.31 ± 0.07 c	13.97 ± 0.22 b	67.60 ± 0.20 d	0.64 ± 0.03 b	20.27 ± 0.31 a
25% DDG	89.21 ± 0.11 c	−0.29 ± 0.03 c	14.29 ± 0.13 a	66.62 ± 0.01 e	0.94 ± 0.04 a	20.49 ± 0.11 a

Note

All data were determined in triplicate. Mean values in the same column followed by the same letter are not significantly different from each other (*p* < .05).

Abbreviations: APF, all‐purpose flour; DDG, distillers dried grain.

### 3.2 Mixolab analysis of dough

Table 5 provides the Mixolab parameters defining dough characteristics, namely changes in water absorption of the dough, peak dough development time, and dough stability. With the increasing amount of DDG in the formula, the water absorption of the dough increased significantly from 53.5% (0% DDG) to 71.4% (25% DDG). Rosell et al. (2011) and Bojňanská, Tokár, and Frančáková (2014) attributed this to increased hydrophilic groups in dietary fiber and greater association with water molecules.

**TABLE 5:** Effects of DDG on Mixolab parameters of dough formulated for steamed bread

Dough properties	Control	10% DDG	15% DDG	20% DDG	25% DDG
Water absorption (%)	53.50 ± 1.00 e	62.03 ± 0.45 d	64.63 ± 0.40 c	68.33 ± 0.06 b	71.40 ± 0.53 a
Development time (min)	1.45 ± 0.10 a	1.18 ± 0.31 ab	1.18 ± 0.06 ab	1.13 ± 0.05 b	1.03 ± 0.07 b
Stability (min)	9.80 ± 0.70 a	9.58 ± 0.37 a	8.38 ± 1.26 ab	7.50 ± 1.58 b	9.38 ± 0.65 ab

Note

Means in the same row with the same letter are not significantly different from each other (*p* < .05).

Abbreviation: DDG, distillers dried grain.

In our study, DDG addition decreased dough development time and dough stability. The development time for control flour (0% DDG) was 1.45 min, which was significantly higher than that of 25% DDG blend. In general, stability decreased as well with increases in DDG. The development time and stability reflect the strength of the protein network structure in the process of dough mixing (Bojňanská et al., 2014). The reduction in dough development time and stability indicated that the addition of DDG weakened the gluten strength, decreased endurance to mixing, and contributed to weakening of the continuous gluten network. DDG addition resulted in gluten dilution and disruption of dough matrix, which in turn affected the ability of dough to stretch and trap expanding gases. Hadnadev et al. (2011) showed that the whole‐grain wheat flour with a higher content of seed coat had lower stability. Bojňanská et al. (2014), however, described that with the increasing addition of potato fiber, the dough development time increased. Tsen et al. (1983) reported that the substitution of DDG in white flour increased water absorption while decreasing the development time and stability time. Wu et al. (2014) noted similar results noted with corn pericarp dietary fiber.

### 3.3 Dough extensibility analysis

The addition of DDG into wheat flour had a significant impact on dough strength and dough extensibility (Table 6). Increasing the level of DDG substitution up to 25% in APF doubled the resistance to extension and reduced the extensibility by one‐half. The addition of DDG reduced the relative content of gluten in wheat flour, which affected the formation and stability of the dough, and decreased the extensibility of the dough. Fu et al. (2014) also pointed out that DDG is rich in rigid dietary fiber that hindered the formation of the gluten network, resulting in the decreased extensibility and the increased stiffness. Wu et al. (2014) and Fu et al. (2015) also observed the detrimental effect on extensibility of dough.

**TABLE 6:** Effects of FDDG substitution on the Kieffer Rig resistance to extension and extensibility of dough formulated for steamed breads

Dough samples	Resistance to extension, g	Extensibility, mm
Control	33.42 ± 1.10 a	21.96 ± 0.99 a
10% FDDG	43.31 ± 0.54 b	16.59 ± 0.25 b
15% FDDG	51.52 ± 0.23 c	14.39 ± 0.46 c
20% FDDG	57.23 ± 0.22 d	12.08 ± 0.28 d
25% FDDG	68.13 ± 0.96 e	10.42 ± 0.39 e

Note

Mean values in the same column followed by the same letters are not significantly different from each other (*p* < .05).

Abbreviation: FDDG, food‐grade distillers dried grain.

### 3.4 Texture analysis of cooked steamed bread

Table 7 provides information on the textural characteristics of steamed bread. With the addition of DDG, the hardness of CSB increased significantly from 450.0 to 3,075.6 g (*p* < .05). Cohesiveness, springiness, and resilience of CSB decreased at the same time. This was mainly due to the gluten content reduction with increasing amount of DDG in the flour. The formation of three‐dimensional network structure in the dough was constrained, resulting in the reduction of the gas cells in the steamed bread, which in turn lead to the increase of steamed bread hardness, and the decrease of cohesiveness, springiness, and resilience (Amir, Hanida, & Syafiq, 2013). Addition of DDG contributed to an increase in adhesiveness owing to the higher moisture content of CSB. As DDG substitutions increased from 0% to 20%, the chewiness of CSB increased from 274.6 to 938.7 g. Lu et al. (2013) also demonstrated that adhesiveness increased with the addition of fiber‐enriched okara in steamed bread while springiness, cohesiveness, and resilience showed reduction.

**TABLE 7:** Effects of DDG on texture analyzer parameters of cooked Chinese steamed bread

Samples	Hardness, g	Adhesiveness, g/s	Cohesiveness	Springiness	Chewiness, g	Resilience
Control	450.02 ± 34.76 e	0.52 ± 0.06 b	0.67 ± 0.01 a	0.90 ± 0.00 a	274.58 ± 23.35 c	0.30 ± 0.00 a
10% DDG	1,235.90 ± 64.89 d	7.39 ± 0.51 b	0.61 ± 0.01 b	0.81 ± 0.02 b	608.02 ± 38.45 b	0.26 ± 0.01 b
15% DDG	2,289.96 ± 24.33 c	28.16 ± 15.24 b	0.54 ± 0.00 c	0.74 ± 0.00 c	918.78 ± 13.44 a	0.23 ± 0.00 c
20% DDG	2,527.48 ± 62.04 b	38.91 ± 17.19 ab	0.53 ± 0.01 c	0.70 ± 0.05 c	938.71 ± 45.98 a	0.22 ± 0.01 c
25% DDG	3,075.60 ± 68.30 a	75.70 ± 48.93 a	0.50 ± 0.02 d	0.57 ± 0.01 d	879.50 ± 48.70 a	0.20 ± 0.01 d

Note

All data were determined in triplicate. Mean values in the same column followed by the same letters are not significantly different from each other (*p* < 0.05).

Abbreviation: DDG, distillers dried grain.

### 3.5 C‐Cell image analysis

Table 8 provides image analyses data of CSB with varied levels of DDG. A decrease in the cross sectional slice area of steamed bread occurred with increasing levels of DDG. This showed that the addition of DDG resulted in a more compact or dense bread. There was also a trend toward a decline in the number of cells in CSB. Cell diameter and cell wall thickness for all steamed breads showed a range 0.38–0.42 mm and 1.56–1.84 mm, respectively. The number of cells and wall thickness are good indicators of proofing quality (O'Shea et al., 2015). Desired CSB crumb structure includes a higher number of cells and smaller values for wall thickness and cell diameter. CSB with higher substitutions of DDG had corresponding lower levels of fermentable carbohydrates for fermentation and yeast action. They yielded lower amounts of leavening gas (carbon dioxide) resulting from the reduced amount of starch and decreased starch fermentation. The decreasing protein network structure resulting from gluten dilution retained less gas resulting in a decline in number of cells. Mäkinen and Arendt (2012) reported that higher levels of barley and oat in bread formulations decreased the number of cells and increased the wall thickness, which deteriorated the quality of bread.

**TABLE 8:** C‐Cell crumb image analysis of steamed bread containing varying levels of distillers dried grains

Samples	Control	10% DDG	15% DDG	20% DDG	25% DDG
Slice area (mm^2^)	4,763.67 ± 68.19 a	3,810.67 ± 52.27 b	3,724.00 ± 78.26 bc	3,642.00 ± 137.01 c	3,403.33 ± 66.08 d
Wrapper length (mm)	280.50 ± 1.41 a	247.93 ± 7.31 b	238.90 ± 0.78 c	230.90 ± 3.74 d	221.03 ± 1.42 e
Number of cells	4,144.33 ± 204.18 a	3,347.00 ± 323.11 b	3,388.00 ± 237.31 b	3,231.33 ± 91.27 bc	2,886.33 ± 89.81 c
Cell diameter (mm)	1.69 ± 0.05 ab	1.71 ± 0.08 ab	1.56 ± 0.13 b	1.60 ± 0.08 b	1.84 ± 0.13 a
Wall thickness (mm)	0.38 ± 0.02 b	0.40 ± 0.02 ab	0.39 ± 0.01 ab	0.40 ± 0.00 ab	0.42 ± 0.01 a
Slice brightness	131.13 ± 0.21 a	96.47 ± 4.10 b	85.60 ± 1.23 c	81.23 ± 0.32 cd	79.27 ± 3.60 d

Note

All data were determined in triplicate. Mean values in the same column followed by the same letters are not significantly different from each other (*p* < .05).

Abbreviation: DDG, distillers dried grain.

### 3.6 Sensory analysis of steamed bread

**FIGURE 3:**
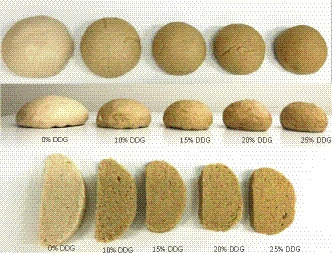
Chinese steamed bread enriched with varying levels of DDG (DDG, distillers dried grain)

Figure 3 provides images of CSB prepared with varying levels of DDG. Table 9 provides the sensory scores of quality analysis of CSB with DDG. With increasing levels of DDG, the specific volumes of steamed bread decreased significantly from 14.75 (0% DDG) to 3.08 (25% DDG). This phenomenon was due to the DDG contribution to gluten dilution and resultant deterioration of gas retaining ability (Abbott, O'Palka, & McGuire, 1991). Specific volume reflects the magnitude of volume expansion of the dough and is, therefore, a desirable trait in steamed breads. Specific volume influenced the elasticity and chewiness of the bread. From Table 9, it is notable that the elasticity and chewiness decreased with high levels of DDG, as did CSB height and stickiness. Amir et al. (2013) demonstrated that higher fiber content increased the water absorption capacity, which, in turn, resulted in a compact loaf structure. Flavor score decreased with increasing substitution of DDG. Addition of DDG to bread imparted a corn aroma, and the higher amount of DDG introduced a bitter taste. Good steamed bread should be chewy and slightly sticky and have good elasticity. When the addition of DDG is 15% or less, the quality score of steamed bread is more than 70, which indicated that the quality of steamed bread was acceptable.

**TABLE 9:** Sensory quality analysis of steamed bread enriched with distillers dried grains

Quality parameter (score)	Control	10% DDG	15% DDG	20% DDG	25% DDG
Specific volume (15)	14.75 ± 0.87 a	11.58 ± 1.24 b	8.75 ± 1.22 c	4.67 ± 0.65 d	3.08 ± 0.79 e
Height (5)	2.75 ± 0.62 a	2.50 ± 0.67 ab	2.50 ± 0.67 ab	2.17 ± 0.58 bc	1.67 ± 0.49 c
Surface color (10)	10.00 ± 0.00 a	7.75 ± 0.62 b	7.00 ± 0.95 c	6.08 ± 0.67 d	4.25 ± 01.36 e
Surface structure (10)	9.67 ± 0.49 a	9.00 ± 0.95 ab	8.00 ± 0.95 bc	7.83 ± 1.47 c	7.08 ± 1.83 c
Exterior appearance (10)	9.17 ± 1.40 a	8.50 ± 1.38 ab	7.92 ± 1.51 ab	8.33 ± 1.15 ab	7.33 ± 1.83 b
Interior structure (15)	13.67 ± 1.87 a	11.83 ± 3.10 ab	12.00 ± 2.59 ab	10.17 ± 2.72 b	10.58 ± 2.71 b
Elasticity (10)	9.75 ± 0.45 a	8.17 ± 1.59 b	7.33 ± 1.61 bc	6.42 ± 2.43 c	4.83 ± 2.21 d
Chewiness (10)	9.58 ± 0.67 a	8.00 ± 0.95 b	7.42 ± 1.44 bc	6.42 ± 1.38 cd	5.92 ± 1.88 d
Stickiness (10)	8.75 ± 1.60 a	7.33 ± 1.50 b	6.75 ± 1.76 b	6.08 ± 1.44 bc	4.75 ± 1.86 c
Flavor (5)	4.50 ± 0.90 a	4.33 ± 0.98 a	3.83 ± 0.72 ab	3.42 ± 0.79 b	3.33 ± 1.23 b
Total score (100)	92.58 ± 3.29 a	79.00 ± 5.20 b	71.50 ± 4.58 c	61.58 ± 6.24 d	52.83 ± 6.91 e

Note

All data were determined in triplicate. Mean ± standard deviation values in the same column followed by the same letter are not significantly different from each other (*p* > .05). Steamed bread quality score: poor = <70 points, average or general = 70–79 points, good = 80–89, and excellent = 90–100 points.

Abbreviation: DDG, distillers dried grain.

Almeida, Chang, and Steel (2013) and O'Shea et al. (2015) reported that the addition of dietary fiber reduced the specific volume and crumb luminosity of bread significantly. Sabanis, Lebesi, and Tzia (2009) observed similar effects of maize fiber on the specific volume. Liu et al. (2011) also demonstrated that the increased content of DDGS darkened bread's appearance. Tsen et al. (1983) found that the replacement at 20% DDG reduced the grain score. Fu et al. (2014) found that the bread with the highest dietary fiber decreased its overall acceptability.

### 3.7 Scanning electron microscopy (SEM)

**FIGURE 4:**
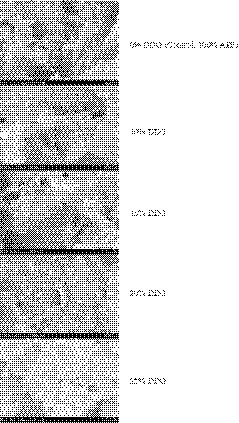
Scanning electron microscopy of Chinese steamed bread enriched with 0%–25% distillers dried grains (APF, all‐purpose flour; DDG, distillers dried grain)

The structure of the gluten network is important for the rheological properties of dough. The microstructure of the gluten matrix can be assessed by using SEM. Figure 4 provides SEM of control and DDG fortified steam bread at a magnification of 3,000×. It is noteworthy that the 0% DDG breads made exclusively with APF had a honeycombed structure with large gas cells. The results indicated the structure of steamed bread with higher amounts of DDG was more compact. Higher amounts of fiber in DDG hindered the formation of gluten network structure and compromised the integrity of gas cells, resulting in low gas retention. Sabanis et al. (2009) reported that bread made with wheat and barley fiber show richly endowed small gas cells, resulting in a dense structure.

## 4 CONCLUSIONS

Distillers dried grain is a low‐cost functional food ingredient owing to its large output as a co‐product of ethanol production (39 million tons/year) as well as its richness in dietary fiber and protein content. At $130 per ton at current prices, DDG is valued at $0.07 per pound and shows potential as an enrichment ingredient. New applications in food products will bring a novel source of protein and dietary fiber into the human food supply while improving the health benefits of traditional food. Modest inclusion of fiber in flour formulations can be tolerated in several dough systems as demonstrated in flat breads and other yeast leavened products if stronger flours are used and if flavor and texture profiles are not undermined. Addition of up to 15% FDDG in dough rheology was possible without significantly compromising texture and taste of CSB. Increased substitution of DDG in APF beyond 15% reduced the gluten content of flour blends. These changes in the composition of blends undermined the formation of gluten network in the dough resulting in an increase of water absorption, decrease of development time, decrease in extensibility, reduction in the number of air cells, and the specific volume of the finished product. Texture of CSB showed that with increasing amounts of DDG, the hardness and adhesiveness of CSB increased while the cohesiveness decreased. Because of natural pigment in DDG, the brightness (L*) of blends and steamed bread decreased and yellowness (b*) increased. The higher substitution (20% and 25%) of DDG in APF caused a significant reduction in CSB scores according to a panel of trained judges. The 10% and 15% DDG substitutions yielded steam breads that were acceptable by a trained sensory panel. CSB formulations are particularly lean, containing only flour, yeast, and water as principle ingredients. Such lean formulations permit modest enrichment levels (up to 15%) without detriment to rheology and food quality. Results from this study demonstrate food applications of DDG and the development of fiber‐rich and protein‐rich steamed bread. Improvement of texture, mouth‐feel, and flavor of steamed bread may benefit from use of sugar and dough improvers in CSB formulation.

## CONFLICT OF INTEREST

The authors declare that they have no conflict of interest to report in this publication.

## ETHICAL APPROVAL

This study conforms to US Guidelines for Human Subjects, and the study protocol was reviewed and approved by the South Dakota State University Institutional Review Board.
